# Clinical Application of Exercise Stress Echocardiography in an Outpatient Pediatric Population

**DOI:** 10.3390/jcm13082191

**Published:** 2024-04-10

**Authors:** Nuno Cotrim, Hugo M. Café, Jorge Guardado, Pedro Cordeiro, Hortense Cotrim, Rui Martins, Luís Baquero, Carlos Cotrim

**Affiliations:** 1 Hospital Distrital de Santarém, 2005-177 Santarém, Portugal; nuno_cotrim1@hotmail.com; 2 Heart Center do Hospital da Cruz Vermelha, 1500-048 Lisboa, Portugal; jobeguardado@gmail.com (J.G.); luisbakero@gmail.com (L.B.); 3 Unidade Cardiovascular, 2350-325 Torres Novas, Portugal; 4 Hospital Particular do Algarve, 8005-226 Faro, Portugalpmcordeiro@hotmail.com (P.C.); 5 Instituto Jean Piaget do Sul, 1950-157 Lisboa, Portugal; hortensecotrim@gmail.com; 6 Faculdade de Ciências de Lisboa, Universidade de Lisboa, 1749-016 Lisboa, Portugal; ruimartins@ymail.com

**Keywords:** exercise stress echocardiography, children, pediatric population, intraventricular gradients

## Abstract

**Background**: Exercise stress echocardiography (ESE) is commonly employed in adults, but its applicability in pediatric populations remains to be clarified. **Methods:** A total of 309 consecutive children (C), with a mean age of 14.1 ± 2.6 years (range 6–17 years), underwent treadmill ESE starting in 2002. They were divided into two groups: Group I comprised 258 children, including 237 with symptoms related to exercise (such as chest pain, fatigue, lipothymia/syncope, or one aborted sudden death), 15 with electrocardiogram (ECG) abnormalities, and 6 with a positive ECG stress test showing ST changes. Group II consisted of 10 asymptomatic children whose parents requested routine screening, 11 with symptoms unrelated to exercise, 12 with a family history of sudden death, and 17 with known pathologies (including 10 with hypertrophic cardiomyopathy, 2 with aortic coarctation, and the remainder with various conditions, such as Cortriatriatum sinister, pulmonary stenosis, subaortic stenosis, bicuspid aortic valve, left ventricular hypertrophy related to arterial hypertension, and aortic switch operation). Regional wall motion abnormalities (RWMAs) and transvalvular or intraventricular (IVG) gradients were assessed using 2D and continuous-wave Doppler, respectively, in all cases. **Results:** The success rate was 100% (309/309). Stress-induced RWMAs were observed in two children. A significant IVG (>30 mmHg) was detected in 101 out of the 258 children (39%) in Group I, who presented with exercise-related symptoms, ECG abnormalities, or positive stress ECG. In Group I, the odds ratio (OR) of ESE reproducing the symptoms in children with IVG compared to those without IVG was 8.22 (95% CI: 4.84–13.99, *p* < 0.001). **Conclusions:** Treadmill ESE is both feasible and safe for pediatric populations. RWMAs demonstrated limited utility in our cohort of children, while IVG induced by exercise was frequently observed in symptomatic children.

## 1. Introduction and Aims

Stress echocardiography, particularly exercise stress echocardiography (ESE), is an imaging technique that has been used by the authors since 1996 [1] to evaluate and study suspected or known cardiac conditions in adult patients. We consider that ESE has the potential to offer the same benefits to the pediatric population; however, it is an underexplored diagnostic tool, perhaps due to concerns regarding the learning curve of the method and its safety and applicability in terms of pediatric age. Given its diagnostic accuracy, ability to assess cardiac function, and lack of radiation, ESE should be considered a first-line diagnostic method, as it is a helpful tool in the clinical assessment of pediatric patients [2,3,4,5,6,7,8,9,10,11,12,13,14]. Physicians need to be aware that, despite the clinical necessity of imaging studies, the associated ionizing radiation exposure could pose an increased lifetime attributable risk of cancer [15]. In children with congenital heart disease, ESE can help reduce the aggressive use of radiation from computed tomography (CT) scans and scintigraphy. This is particularly important since a child aged between 15 and 20 years old, with congenital heart disease, has already accumulated a dose of radiation per exposure that is equivalent to 2000 chest X-rays [16]. Children with heart disease, whether congenital or acquired, are a vulnerable population group from an ethical perspective. This is because they require ongoing medical care, which places a greater responsibility on health professionals, particularly doctors. The reason for this increased responsibility is that children lack the full capacity to make decisions and do not have adequate knowledge about the different options available in order to choose the most appropriate healthcare. Health professionals are responsible for making protective decisions for the well-being and lives of their patients, including choosing the most suitable diagnostic and therapeutic methods that will have both immediate and long-term effects on their patients’ quality of life [17]. It is important to rigorously justify radiological procedures in any situation involving ionizing radiation, with a preference for non-ionizing imaging modalities. The basic objective of optimizing radiological protection is to adjust image parameters and implement protective measures so that the required image is obtained with the lowest possible radiation dose while maintaining sufficient quality for diagnostic interpretation [18]. Regarding pediatric imaging, the main aspect is the capacity of the equipment used, which should allow for an adaptation of radiation dose to the child’s size and weight. In conclusion, it is worth noting that, after becoming aware of the risks associated with exposure to ionizing radiation throughout life and despite recognizing the vital role, both diagnostic and therapeutic, of medical imaging procedures, it is only prudent to perform such procedures using the lowest possible radiation doses while ensuring their diagnostic value [19].

Conventional health assessments are typically conducted while the patient is at rest. However, ESE expands this evaluation by gathering data in a physiological context that more closely simulates the active state commonly experienced by children. This method enables the assessment of myocardial perfusion in children suspected of having coronary artery disease, as well as the examination of cardiac gradients or functional reserve in patients with non-coronary artery issues. It is imperative that such studies are performed by a trained cardiologist [1] or sonographer under direct medical supervision. Stress can be induced in children through either exercise or pharmacological means. The recommendations of the European Association for Cardiovascular Imaging and the American Society of Echocardiography highlight that exercise is the stressor of choice for most indications, reinforcing that any patient capable of physical exercise must be tested with an exercise modality, as this preserves the integrity of the electromechanical response and provides valuable information about their functional status. Performing echocardiography during exercise also makes it possible to establish links between symptoms, cardiovascular workload, wall motion anomalies, and hemodynamic responses, such as pulmonary pressure, flows, and transvalvular gradients [20]. Echocardiography is employed to identify regional wall motion abnormalities when assessing myocardial perfusion or gradients and to evaluate function when examining children without coronary artery disease. Conditions warranting stress echocardiography, which may involve potential coronary artery pathology, include Kawasaki disease [13], transplant graft vasculopathy, arterial switch operation for the transposition of the great arteries, anomalous coronary artery origins or courses, hyperlipidemia, diabetes mellitus, and supravalvular stenosis. Additionally, stress echocardiography aids in our understanding of transvalvular or intraventricular gradient behavior during physical activity in conditions such as hypertrophic cardiomyopathy or aortic and pulmonic stenosis, cardiac pressures in pulmonary hypertension, and ventricular function in conditions like dilated cardiomyopathy or mitral and aortic regurgitation [1,2,3]. In the assessment of children with confirmed or suspected cardiac issues, stress echocardiography, especially exercise stress echocardiography, should be seriously considered. We further furnish crucial insights by meticulously detailing cardiovascular anatomy and physiology, as well as uncovering some unexpected findings, such as intraventricular gradients induced by exercise [1]. Most diagnostic examinations, such as computed tomography and cardiac magnetic resonance, are conducted with children at rest. However, as children are typically active and engaged in various activities, they often experience symptoms that may be indicative of heart disease. It is during physical exertion that most adverse cardiac events occur, and it is also during this time that more subtle or concealed pathologies can be revealed, potentially providing an opportunity for intervention before complications arise. Understanding cardiac function during exercise, when symptoms frequently manifest, is essential for devising effective management strategies.

Our impulse to perform ESE in children was initiated following requests from pediatricians, pediatric cardiologists, general practitioners, and cardiologists who assess children in their clinical practice and refer them for ESE. The present study, undertaken by cardiologists specialized in the care of adult patients with significant experience in exercise echocardiography, is intended to present their experiences in the clinical application of ESE in children between 2002 and 2019.

## 2. Materials and Methods

This study was approved by the Ethics Committee of the Heart Center of the Red Cross Hospital in Lisbon. All children that underwent an ESE performed by our team at Red Cross Hospital, Ucardio, and Hospital Particular of Algarve were found by reviewing the internal database of EchoLabs. The medical records were reviewed for patient data collection, namely demographics, clinical diagnosis, the indications for ESE, and test results.

Written informed consent from parents or tutors and written informed assent were obtained from all subjects involved in this study [21].

**Figure 1:**
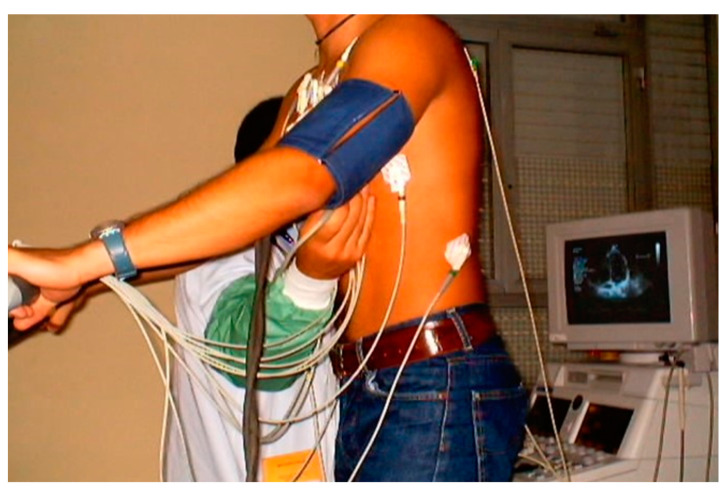
Image acquisition in the orthostatic position during exercise stress echocardiography using a treadmill for a 17-year-old boy with chest pain related to exercise.

Children underwent treadmill exercise tests guided by symptoms using the standard Bruce protocol [22]. A modified version of the Bruce protocol was utilized to facilitate the assessment of Doppler parameters, providing a simpler evaluation compared to the classical Bruce protocol. Standard twelve-lead electrocardiographic monitoring was conducted, with measurements of ST-segment alterations and any arrhythmias, heart rate, and blood pressure taken at baseline and each stage of the exercise protocol. Before the exercise test, a baseline echocardiogram was performed with the child in the left lateral decubitus position for initial assessment. This involved acquiring 2D and M-mode images in at least four planes: parasternal long- and short-axis and apical four- and two-chamber views (with Doppler parameters evaluated and stored based on clinical requirements). During the exercise test, two-dimensional echocardiographic images were obtained from the parasternal long- and short-axis and apical four- and two-chamber views, with the child in a standing position at rest, during exercise (see Figure 1), at peak exercise, in the immediate post-exercise period, and during recovery [1].

Image acquisition during peak and immediately post-exercise was facilitated using a continuous image-capturing system. Subsequently, frames showcasing optimal image quality and prominent Doppler signs were meticulously chosen in each perspective. The obtained digitized images underwent thorough review and comparison, presented in a digital side-by-side quad-screen format within the echocardiographic equipment. Following the exercise test, as appropriate, patients were promptly positioned in the left lateral decubitus posture, and images were reacquired in identical planes. In circumstances where standing posture was pertinent, such as for detecting and evaluating intraventricular gradients (IVGs) in cases of hypertrophic cardiomyopathy (HCM) or symptomatic children, subjects were instructed to remain standing post-stress test, facilitating echocardiography in this stance [23] (Video S1).

We registered 309 successive children (Table 1) referred by assistant physicians, including general practitioners, pediatricians, pediatric cardiologists, and cardiologists. Their average age was 14.1 ± 2.6 years (ranging from 6 to 17 years), and they underwent treadmill ESE using the previously mentioned methodology between 2002 and 2019.

The children were categorized—for analytical purposes—into two groups: Group I consisted of 258 children, among whom 237 exhibited symptoms related to exercise (such as chest pain, fatigue, lipothymia/syncope), and 1 experienced aborted sudden death following participation in a triathlon [23]. Additionally, 15 children showed resting ECG alterations, and 6 tested positive according to the electrocardiographic criteria elicited by stress testing using a treadmill. Group II comprised 10 asymptomatic children whose parents sought an examination for routine screening, 11 with symptoms unrelated to exercise, and 12 with a family history of sudden death. Furthermore, 17 children had known pathologies, including 10 diagnosed with hypertrophic cardiomyopathy, 2 with aortic coarctation, and the remaining with Cortriatriatum sinister, pulmonary stenosis, subaortic stenosis, bicuspid aortic valve, left ventricular hypertrophy related to arterial hypertension, and aortic switch operation. All children underwent regional wall motion abnormalities (RWMAs) and continuous-wave Doppler assessments (transaortic, transvalvular, or intraventricular gradients).

## 3. Results and Statistical Analysis

**Figure 2:**
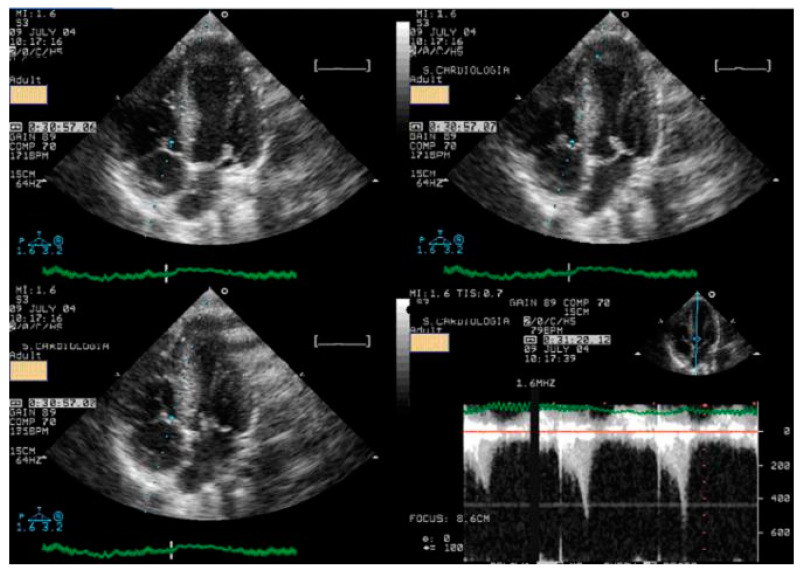
Significant intraventricular gradient in a young boy with angina and ST alterations in the exercise stress test. He also developed systolic anterior movement (SAM) of the mitral valve [1].

**Figure 3:**
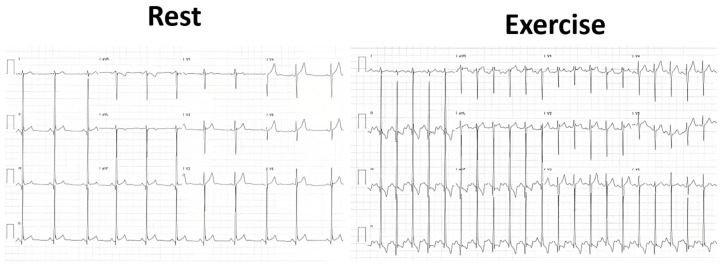
Significant ST alterations in the exercise stress test of one child with severe angina and intraventricular gradient with SAM of the mitral valve during the ESE.

Statistical analysis was performed using the SPSS IBM Program 24 Statistics (New York, NY, USA). The summary measures are presented as mean ± standard deviation for continuous variables and counts, with percentages for categorical variables. The success rate of the methodology was 309/309 (100%). Stress-induced RWMAs were present in only two children, one with HCM and the other with normal coronary arteries (a coronary computed tomography angiography (CCTA) was performed that revealed no obstructive epicardial coronary artery disease). A significant orthostatic exercise-induced IVG (Figure 2) (>30 mmHg) was present in 101 (39%) of the 258 children evaluated due to exercise-related symptoms, alterations in the ECG, or positive stress ECG (Figure 3) and considered as candidates for beta-blocker therapy [1]. 

**Figure 4:**
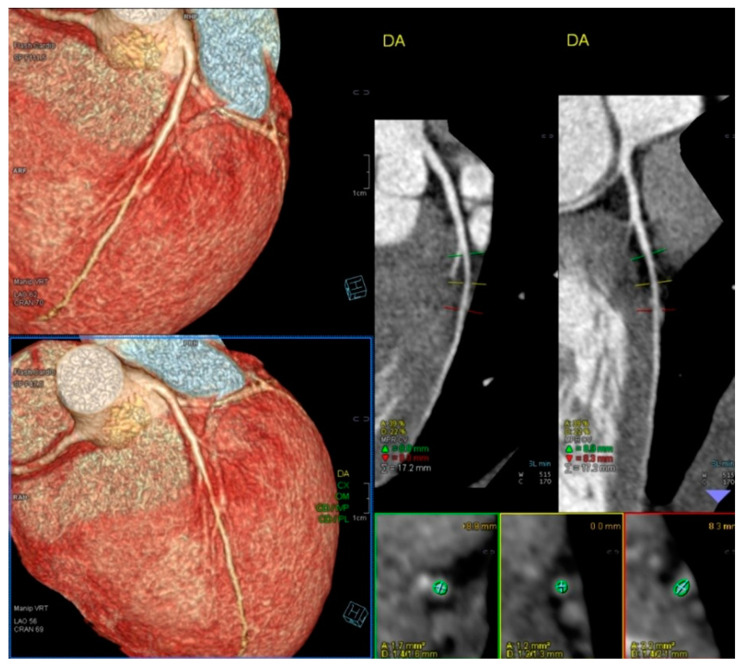
CCTA of a 13-year-old boy with chest pain followed by syncope related to intense exercise (playing rugby). A significant increase in troponin was detected after this episode.

**Figure 5:**
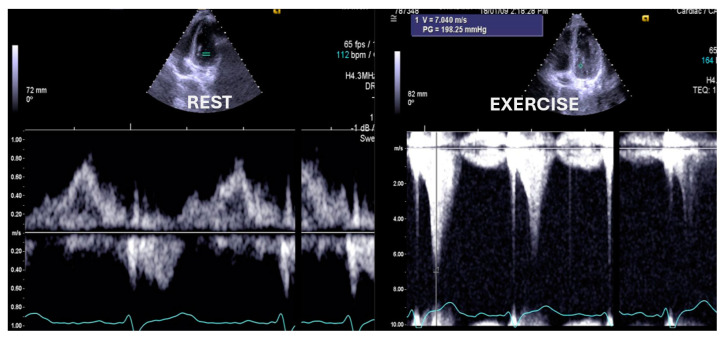
Significant intraventricular gradient in a young boy with angina and syncope during strenuous exercise.

A comparison between the two groups defined by the variable IVG was performed by employing Welch’s test for two independent samples because the sample sizes were unequal. The size of our sample allows us to resort to the central limit theorem, implying that we do not have to verify the normality of the distributions. In Group I, children who developed IVG attained a greater heart rate (HR) of 184 ± 12 vs. 174 ± 16 (*p* < 0.001) and a higher systolic blood pressure (BP) of 150 ± 19 mmHg vs. 136 ± 23 mmHg (*p* < 0.001). To compare the odds of reproducing symptoms across the groups defined by IVG, we performed a logistic regression, where the response variable was “Reproduce Symptoms”, and the independent variable was IVG. The OR of exercise stress echocardiography to reproduce the symptoms these children had when comparing the 101 children with IVG with the 158 without IVG was 8.22 ((4.84–13.99) *p* < 0.001 (95% CI)). One of the children who suffered from chest pain and syncope related to intense exercise had an increase in troponin, but both their coronary angiography and CCTA were normal (Figure 4). After those exams, an ESE was performed, and a significant IVG without SAM of the mitral valve was seen (Figure 5).

Nine other children with chest pain and ST alterations related to exercise performed CCTA that excluded obstructive epicardial coronary artery disease. Finally, eight children without IVG had exertional asthma. The diagnosis was performed during ESE with lung auscultation during and/or at the end of the exam. 

**Figure 6:**
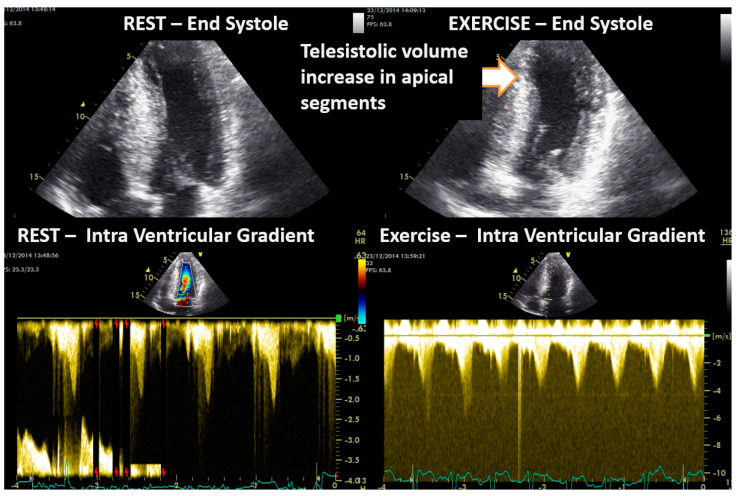
Intraventricular obstruction and contractility alterations during exercise in one symptomatic 16–year-old boy with obstructive hypertrophic cardiomyopathy.

In Group II, the ten children with a diagnosis of HCM were non-obstructive before exercise, and four developed intraventricular obstruction with exercise. One of these four children is a symptomatic young boy whose exercise-induced obstruction was the first sign of HCM, having no hypertrophy at the moment of the ESE [24]. Another developed dilation of the left ventricle, particularly at the level of the apical segments (Figure 6). 

**Figure 7:**
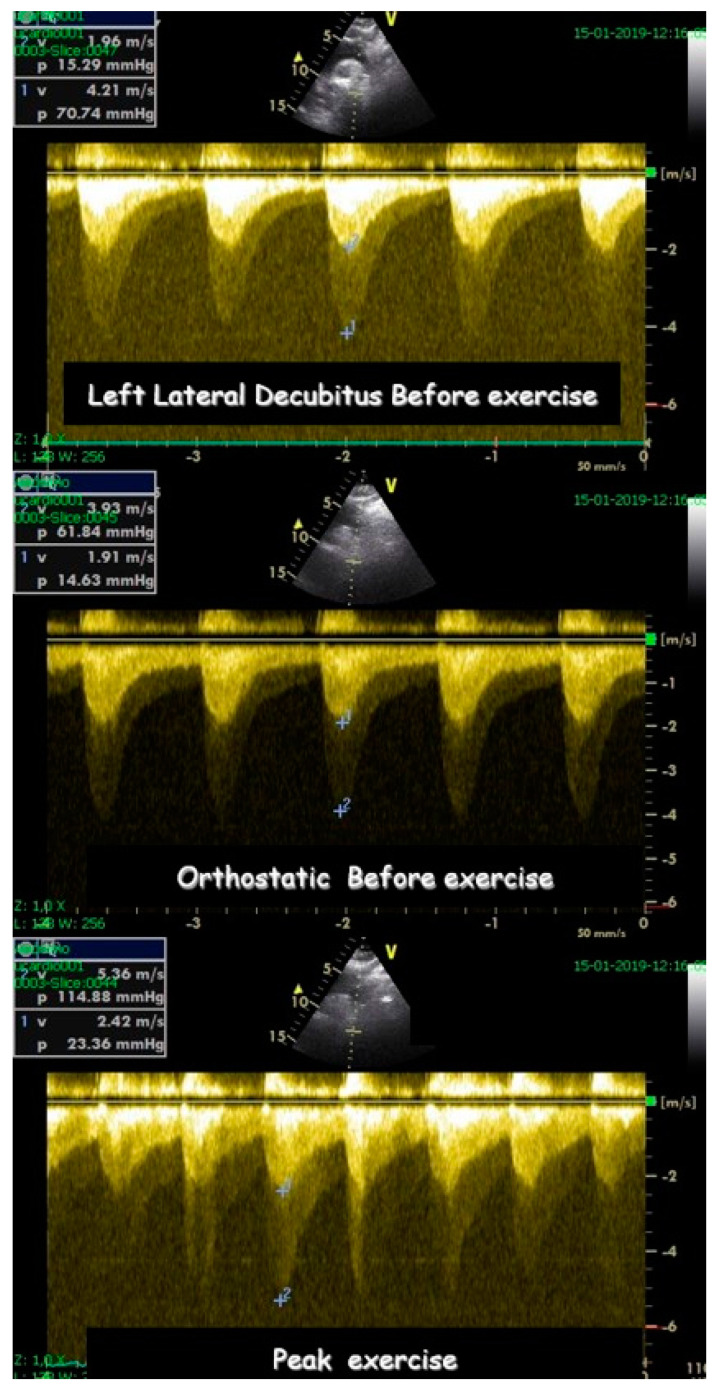
Aortic gradient evaluated in a patient with aortic coarctation previously treated with a stent. Based on the exercise stress echocardiography results, the child was treated again [1].

The ESEs for the 10 asymptomatic children whose screening was requested by their parents, 11 with symptoms unrelated to exercise, and 12 with antecedents of sudden death in the family showed normal results. The two children with previously intervened aortic coarctation revealed an increase in the aortic gradient, with reintervention required in one of them (Figure 7).

**Figure 8:**
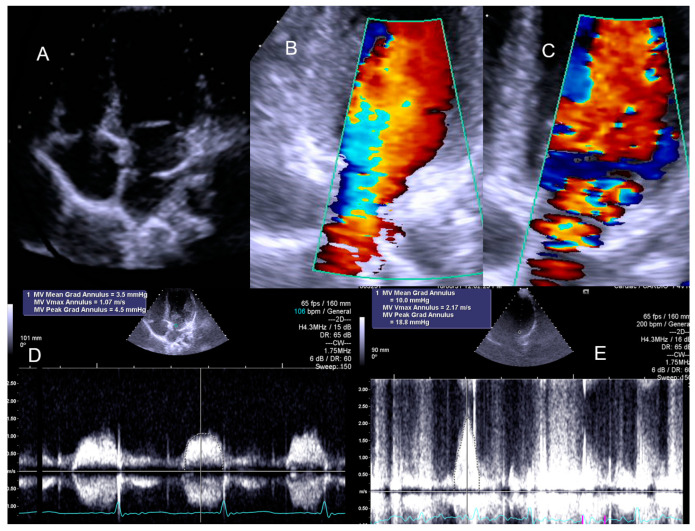
The exercise Doppler data in conjunction with the exercise and clinical data led the medical team to keep the patient in close clinical follow-up. (**A**): Intra–atrial septum in “cortriatriatum”; (**B**): color flow before exercise; (**C**): color flow at peak exercise; (**D**): continuous–wave (CW) flow before exercise; (**E**): CW flow at peak exercise [1].

The child with Cortriatriatum sinister revealed a nonsignificant increase in the diastolic mean gradient (Figure 8). 

The children with pulmonary stenosis, subaortic stenosis, and bicuspid aortic valve revealed non-significant increases in the systolic mean gradient. The children with left ventricular hypertrophy related to severe arterial hypertension developed a significant intraventricular gradient without SAM of the mitral valve. The child with an aortic switch operation was negative for ischemia.

## 4. Discussion

**Figure 9:**
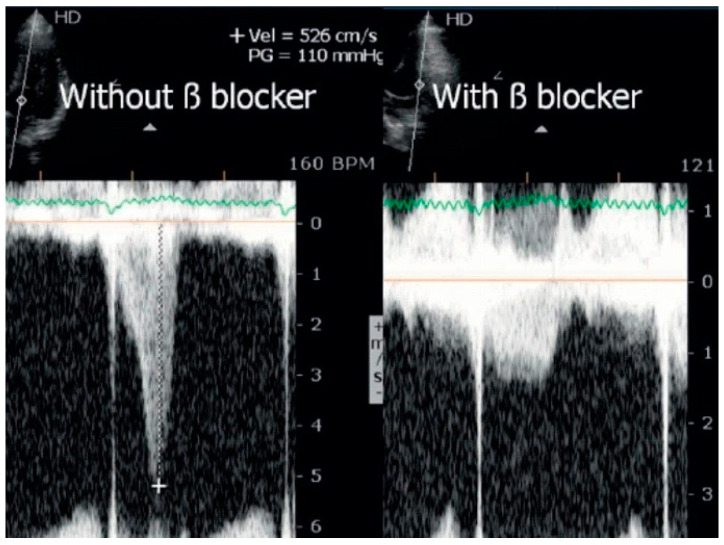
Intraventricular gradient in a child assessed before and on beta-blocker therapy [39].

In the evaluation of children with known or suspected heart disease stress, echocardiography is underused when compared to the adult population. For adults, there are clear guidelines that should be followed for the proper use of stress echocardiography [25,26,27,28]. On the other hand, there are no clear recommendations regarding the use of ESE in the pediatric population, and some of those who consider the use of stress echo focus on the limitations [28] instead of the advantages, such as being free of radiation, medications, and iodine-based contrast. From an ethical standpoint, the utilization of CT scans and other diagnostic procedures involving radiation in pediatric patients warrants careful consideration. While these procedures offer significant medical benefits, they also carry inherent risks, notably the potential for radiation-induced cancer. Concerns arise particularly in children due to their heightened radiosensitivity and the possibility of higher radiation doses [29]. In accordance with this concern, obtaining informed consent from both parents and children becomes imperative, depending on the latter’s age and comprehension abilities. This consent should follow a comprehensive discussion, including details regarding the recommended diagnostic examination, its associated risks and benefits, as well as alternatives. Research indicates that children aged 10 and older typically possess sufficient understanding to provide assent when presented with relevant information [16]. Additionally, discussions should cover alternative diagnostic methods and their associated risks, as well as the potential risks of forgoing the examination [30]. Numerous studies indicate that CT scans in children may result in cumulative radiation doses of approximately 50 mGy, potentially tripling the risk of leukemia, while doses around 60 mGy may similarly elevate the risk of brain cancer. Despite the relatively low absolute risks of these cancers, they remain significant. For instance, within a decade following the first scan in children under 10, an estimated excess of one case each of leukemia and brain tumor per 10,000 head CT scans may occur [31]. The aforementioned authors stress the importance of ensuring that the clinical benefits of CT scans outweigh these small absolute risks. It is advised to keep radiation doses from CT scans as minimal as possible, with consideration given to alternative procedures that do not involve ionizing radiation, where appropriate. In the present study, we show the experience of a group of “adult” specialized cardiologists with significant experience in this field (more than a mean of 500 stress echocardiograms every year during the last 20 years (1760 exercise stress echocardiograms in 2023) performing exercise stress echocardiography in a group of children. The evaluation of chest pain related or not to exercise in pediatric age, beyond clinical evaluation, is usually studied with an electrocardiogram, exercise stress test [32], transthoracic echocardiogram, 24 h Holter, and, more recently, with exercise stress echocardiography, nuclear medicine, cardiac magnetic resonance, and coronary computed tomography angiography [1,33]. A significant effort was made in the last twenty years to attain a rational use of resources (in this case, exams to study children with symptoms) [34,35,36,37]; for example, the exercise stress test (without echo) rarely helps to make conclusions about the etiology of symptoms, such as chest pain, presented by children. We underline that beginning the diagnostic approach for children presenting with symptoms related to exercise with ESE permits a reduction in the use of exams with radiation [1,2] and also defines the proper treatment in a significant number of them, as exemplified by the boy in Figure 4 and Figure 5 and the boy in Figure 9 with the use of beta-blockers [38,39].

We also underline the fact that the ESE performed by a cardiologist with significant clinical experience permitted the reliable diagnosis of effort asthma in eight children. Comparing our study with others in the literature [2,3,4,5,6,7,8,9,10,11,12,13,14], we found significant differences in methodology, as we only utilized exercise stress echocardiography on the treadmill with permanent clinical and echocardiographic evaluation (Video S1) [1]. We looked for usual details, like RWMAs to exclude coronary disease, valvular gradients, and intraventricular gradients in HCM children; however, the evaluation of intraventricular gradients is also performed in other children by routine, including those with a normal echocardiogram. The detection of exercise-induced intraventricular gradients (IVGs) related to symptoms such as angina, tiredness, or syncope in patients with normal echocardiogram (no hypertrophy) has previously been published [1,16,40,41,42,43,44,45,46]. The association of IVGs with symptoms [1,23,24,45,46] in terms of pediatric age has been previously demonstrated, and our results, where a significant orthostatic exercise-induced IVG appeared, seem to confirm this association. The presence of IVG was considered a normal finding by only one group of investigators [40]. The results of the present study and those from other authors suggest a clear relation between IVG and symptoms [16,40,41,42,43,44], and the clinical response to beta-blocker treatment [38,39] favors this conclusion. Guidelines recommend the use of stress echocardiography in adults when available as a first-line test instead of an exercise stress test. According to our results [1,25,27,47], echocardiography in association with stress ECG should be considered in the pediatric population, since stress ECG alone may be normal or provide a false-positive result (Figure 3) in most children, as noted in our population. Also taking into account safety reasons [48], the results of our study with exercise stress echocardiography, as well as scientific recommendations [20,25,26,27,28,47], ESE should be performed if possible to clarify unexplained exercise-related symptoms.

## 5. Limitations of the Study

Our study has several limitations. First, the fact that it is a retrospective analysis introduces potential bias. Furthermore, the process of data collection is challenging, as it depends on the quality of the clinical evaluation and registration data process. Second, most of the children were outpatients, and we had a small number of congenital pathologies to analyze in this study. As a consequence, we are not able to draw firm conclusions about the use of ESE in this field. Lastly, we only studied ten children with no detected pathology, which made it statistically impractical to consider them as a control group.

## 6. Conclusions

The present study was based on the use of exercise stress echocardiography in children. It was performed by an experienced stress echocardiography team with one hundred percent feasibility and no complications, allowing us to make conclusions useful for clinical decisions in a great number of children. 

Our analysis of this group of children informs us that if we do not search for intraventricular gradients, we will not find them, losing information that may give us a clinical solution for this health problem in children. Taking into account the fact that 39% of the children in the study has exercise-related symptoms, the presence of alterations in the ECG, or developed exercise-induced intraventricular gradients in the exercise stress test, we consider that the lack of useful information from the simple exercise stress test for a medical decision can damage the health of the children and negatively impact the quality of care. For this reason, we propose that the diagnostic flowchart begins preferentially with ESE.

Our results also reveal that ESE can be tailored to every particular clinical situation, such as aortic coarctation, Cortriatriatum, valve stenosis, or hypertrophic cardiomyopathy.

Lastly, we believe that this study exemplifies a way to increase the utilization of stress echocardiography in the pediatric population, mainly through cooperation between cardiologists specialized in the care of adult patients and other specialties caring for children.

## Figures and Tables

**Table 1:** Clinical and demographic characteristics of children undergoing ESE.

Variables	Range	Value
Age	(6 to 17 year)	14.13 ± 2.57
Male		199 (64.4%)
Female		110 (35.4%)
Exercise symptoms are indication to ESE		236 (76.4%)
Abnormal ECG is motive for ESE		16 (5.2%)
Positive exercise stress ECG is the motive for ESE		6 (1.9%)
Other motives for ESE		51 (16.5%)

## Data Availability

The data that support the findings of this study are available from the corresponding author upon reasonable request.

## Supplementary Materials

The following supporting information can be downloaded at https://www.mdpi.com/article/10.3390/jcm13082191/s1: Video S1: Methodology of Exercise stress echocardiography in one child in this study.
